# In Vivo Structural and Functional Abnormalities of the Striatums Is Related to Decreased Astrocytic BDNF in *Itpr2^−/−^* Mice Exhibiting Depressive-Like Behavior

**DOI:** 10.1155/2020/8830670

**Published:** 2020-09-01

**Authors:** Shanmei Zeng, Kai Liu, Jingyu Zhang, Chunhui Chen, Yihua Xu, Yulan Wu, Yanjia Deng, Xuegang Sun, Ge Wen, Linlin Jing

**Affiliations:** ^1^NanFang Hospital, Southern Medical University, Guangzhou, China; ^2^The Second Affiliated Hospital of Guangzhou University of Chinese Medicine, Guangzhou, China; ^3^Integrated Hospital of Traditional Chinese Medicine, Southern Medical University, Guangzhou, China

## Abstract

**Background:**

Previous researches indicate that *Itpr2*^−/−^ mice (inositol 1,4,5-trisphosphate receptor type 2 knockout mice) show depressive-like symptoms; however, little is known regarding the *in vivo* neurobiological effect of Itpr2 as well as the specific pattern of brain abnormalities in *Itpr2*^−/−^ mice. *Methods/Materials*. First, behavioral tests, structural magnetic resonance imaging (MRI), and resting-state functional MRI were performed on *Itpr2*^−/−^ mice and matched healthy controls. Voxel-based morphometry and seed-based voxel-wise functional connectivity (FC) were, respectively, calculated to assess the gray matter volume and the functional activities of the brain in vivo. Second, the sample of relevant changed brain regions was extracted to detect the expression of BDNF. Finally, to further validate the relationship between Itpr2 deficiency and the observed brain abnormalities, we performed Western blotting to detect the expression of pro-BDNF and mBDNF in *Itpr2*^−/−^ C8-D1A (a type of astrocyte).

**Results:**

Compared with controls, *Itpr2*^−/−^ mice showed depressive-like behaviors as well as significantly lower gray matter volume in striatums mainly, periaqueductal GM, and the right frontoparietal cortices as well as lower striatal-hippocampal and striatal-right parietal cortex (mainly for the primary and secondary somatosensory cortex) FC. Moreover, decreased expression of mBDNF was found in both sample tissues of the striatum in *Itpr2*^−/−^ mice and *Itpr2*^−/−^ C8-D1A.

**Conclusion:**

By combining biochemistry and MR analyses, this study provides evidences to support that the Itpr2-related neuropathological effect is possibly mediated by the striatal abnormality associated with dysfunctional astrocytes in *Itpr2*^−/−^ mice *in vivo*, thus may help us better understand underlying mechanisms of Itpr2 deficiency as well as its relation to depressive-like behavior.

## 1. Introduction

Inositol 1,4,5-trisphosphate receptor type 2 (IP_3_R_2_) is a calcium channel receptor located in the endoplasmic reticulum, which is mainly expressed in astrocytes other than other types of cell in the brain [1]. It is considered to be necessary to maintain the normal function in astrocytes especially the exocytosis by which BDNF and ATP are released to extracellular matrix [2]. In fact, a series of neuropsychological abnormal states have been found in *Itpr2^−/−^* mice [3, 4]. Notably, previous studies have found that *Itpr2^−/−^* mice exhibited depression-like behaviors which are considered to be attributed to deficiencies in astrocytic ATP release [5].

There are a lot of research that indicates structural brain abnormalities in major depressive disorder (MDD) [6, 7], while the mechanisms of these abnormalities remain unclear. *In vivo* magnetic resonance imaging (MRI) studies have exhibited that particular areas involved in emotional regulation, such as the hippocampus, amygdala, cingulate cortex, basal ganglia, and prefrontal cortex (PFC) may go through structural changes in MDD patients [8, 9]. Functional magnetic resonance imaging (fMRI) is one of the most important tools for studying the *in vivo* brain activity and anatomy [10], and functional connectivity is one of the most common analysis ways of fMRI [11, 12]. Both the structural and functional abnormalities in depression have been found in a series of brain regions.

Astrocytes can release a variety of neurotrophins under normal conditions [13]. Previous studies indicate that the reduction of neurotrophins, especially brain-derived neurotrophic factor (BDNF), which are the most widely distributed neurotrophin in the CNS and provided by astrocytes mainly in the brain, is a mediator involved in neuronal survival and plasticity of dopaminergic, cholinergic, and serotonergic neurons[14]. BDNF are involved in the pathogenesis of depression, with decreased growth and survival of neurons, and thus possibly leads to gray matter atrophy shown on magnetic resonance imaging [15–17]. BDNF is synthesized as a precursor called proBDNF, which is proteolytically cleaved to generate mature BDNF [18]. Many previous studies have demonstrated stress could decrease the expression of brain-derived neurotrophic factor (BDNF) in limbic structures including the amygdala, hippocampus, and prefrontal cortex. Furthermore, the reduction of BDNF could contribute to the atrophy of certain limbic structures [19], and the stress-induced reduction in BDNF may result in brain tissue loss [10].

To date, little is known about the *in vivo* neurobiological effect of Itpr2 as well as the specific pattern of brain abnormalities in *Itpr2*^−/−^ mice. Based on existing findings, we hypothesize that *Itpr2*^−/−^ mice have the tendency of depressive-like behavior and can be observed with specific structural and functional abnormalities in the brain *in vivo*, which are possibly associated with decreased BDNF levels produced by astrocytes owing to the effect of Itpr2 deficiency. To this end, we combined animal magnetic resonance imaging techniques and biochemical experimental methods to test the hypothesis in this study.

## 2. Materials and Methods

### 2.1. Mice

*Itpr2* ^−/−^ mice were generated by crossing germline-heterozygous-null mutant *Itpr2*^+/-^ mice, which was a gift by Prof. Tian-Ming Gao [5]. The offspring were genotyped by PCR using mouse tail DNA and wild-type (5′-GCTGTGCCCAAAATCCTAGCACTG-3′; 3′-CATGCAGAGGTCGTGTCAGTCATT-5′) and mutant allele-specific primers (neospecific primer 5′-AGTGATACAGGGCAAGTTCATAC-3′; 3′-AATGGGCTGACCGCTTCCTCGT-5′). The PCR products were visualized with ethidium bromide staining.

### 2.2. Cell

C8-D1A (astrocytic type I clone, GFAP positive) was cloned from mice cerebellum [20]. Cells were maintained in Dulbecco's modified Eagle medium (DMEM; Biochrome, Berlin, Germany) containing glucose, 10% heat-inactivated fetal calf serum (FCS; Sigma, St. Louis, MO), and 1% L-glutamine (Gibco, Auckland, New Zealand). *Itpr2*^−/−^ C8-D1A was constructed by lentivirus transfection knocking down protein expression of IP_3_R_2_, and the target sequence was GCCCAGAAGCAATACTGGAAA.

### 2.3. Behavioral Test

#### 2.3.1. Sucrose Consumption Test

Sucrose Consumption Test was conducted on *Itpr2*^−/−^ mice (*n* = 28) and healthy controls (*n* = 20). Two bottles were placed in each cage simultaneously, both of which contained 1% sucrose water 25 ml, and the mice could drink freely for 24 h. Then, one bottle contained 1% sucrose water 25 ml with the other one containing 25 ml of tap water. Mice were given free choice for 24 h. After that, the test was conducted by providing the mice with a free choice between the two bottles (one bottle containing a 1% sucrose solution and the other bottle containing tap water) for 2 h. The consumption of sugar water and tap water was calculated simultaneously in both groups by weighing the bottles containing the liquids. The preference for sucrose was calculated according to the percentage of consumed sucrose solution with respect to the total amount of liquid consumed.

#### 2.3.2. Tail Suspension Test (TST)

TST was implemented on *Itpr2*^−/−^ mice (*n* = 28) and healthy controls (*n* = 20). This experiment referred to the experimental model established by Steru et al. [21]. Briefly, the mice were acoustically and visually isolated and suspended 60 cm above the floor in an inverted position by sticking a medical adhesive cloth on roughly 2 cm of the tail of the mouse about 2 cm. The time during which the mice remained immobile was determined during a test period of 6 min. Mice were considered immobile only when the mice gave up any struggle and remained motionless.

#### 2.3.3. Forced Swim Test (FST)

FST was implemented on *Itpr2*^−/−^ mice (*n* = 28) and healthy controls (*n* = 20). This test was carried out according to the method described by Porsolt et al. [22]. Each mouse was placed in a cylindrical container (diameter, 10 cm; height, 25 cm) filled with water up to 9 cm at 22 ± 1°C. The immobility time was recorded during the last 4 min of the 6-min testing period. Immobility time was defined as the absence of struggle and only slight body movements keep its head afloat.

#### 2.3.4. Open-Field Test (OFT)

The OFT experiment was carried out on 16 control mice and 28 *Itpr2*^−/−^ mice. The voluntary movement and behaviors of the mice were evaluated using the OFT. The OFT box is a square box with the following dimensions: height, 35 cm; length, 60 cm; and width, 60 cm, which is divided into 4 quadrants; each quadrant is divided into 36 equilateral quadrants. The experiment was conducted in a quiet laboratory room under 60 watts of light. Each mouse was gently placed at the center of the square box and observed at 5 min intervals. The apparatus used was wiped with 75% ethanol and dried before each mouse was tested. Motion detection software (EthoVision 7.0; Noldus, Wageningen, The Netherlands) was used to record the center time (CTRTIME) for each mouse.

### 2.4. Magnetic Resonance Scanning

All MRI scans were performed on 14 control mice and 15 *Itpr2*^−/−^ mice using a 7T Bruker scanner (Pharmascan 70/16 US) equipped with a 86 mm birdcage transmit-only RF coil and a receive-only quadrature surface coil. Before scanning, the mice were anesthetized with 3% isoflurane and then mechanically ventilated with 1-1.5% isoflurane. During the examination, the mice were placed on a plastic cradle with the head fixed with a tooth bar and plastic screws in the ear canals. A water circulation system was used to maintain the body temperature of the animals. The level of anesthesia was monitored, and the respiratory rate was kept above 60 breaths per min. For the structural MRI, a 3D T2-weighted images (3D-T2WI) were scanned using a Turbo RARE sequence with the following parameters: TR/TE = 1800/45, flip angle = 90°, matrix = 256 × 256, voxel size = 0.078 × 0.078 × 0.512 mm, 32 slices, and slice thickness/gap = 0.512/0 mm. For the resting-state fMRI, a spin-echo echo-planar imaging (SE-EPI) sequence was used with 500 time points, TR/TE = 1500/21.2 ms, flip angle = 90°, matrix = 96 × 96, voxel size = 0.156 × 0.156 × 0.7 mm, 15 slices, and slice thickness/gap = 0.7/0 mm.

### 2.5. MRI Preprocessing and Analysis

Prior to preprocessing, all the structural and functional MRIs were resized by a factor of 10. The 3D T2-WI images of all the mice were processed using the SPM12 software (Wellcome Department of Cognitive Neurobiology, University College of London, UK) for VBM analysis. First, the images were linearly registered (12-parameter affine) to approximate the brain space of C57Bl6, which is a previously established template specific for the brain anatomy of C57 mice [23]. Thereafter, the structural images were segmented into GM, white matter, and cerebrospinal fluid images based on the tissue probability maps provided with the C57Bl6 template. Then, based on the segmentation results, the GM images were spatially normalized to the template, and then Jacobian modulated. Finally, the resultant GM images were smoothed using an 8 mm FWHM Gaussian kernel.

For the analysis of rs-fMRI, the first 10 volumes of resting-state scans were discarded. The resulting 490 volumes were corrected for slice timing and for the head motion (a least-squares approach and a six-parameter spatial transformation). Then, the brain fMRIs were spatially normalized to the template in accordance with the transformation established by the normalization of 3D T2-WI images, with the voxel size resampled into 4 × 4 × 4 mm^3^. Thereafter, an 8 mm FWHM Gaussian kernel was used to smooth the fMRIs, and voxel-wise detrending, filtering with a bandpass filter of 0.01-0.08 Hz, and regression of the white matter signal, cerebrospinal fluid signal, mean global signal, and head motion (Friston 24) was done successively.

Calculation of the seed-based FC maps was done as follows. First, the seed region of interest (ROI) was selected by referring to the VBM results as well as the previously established atlas of the mouse brain [24]. Second, the whole-brain resting-state FC map was created by calculating the Pearson correlation coefficient between the resting-state time series extracted from the seed ROI and the time series from all other brain voxels. Here, a Fisher Z-transformation was done to increase the normality of FC data, and the calculation was restricted to positive correlation.

### 2.6. Immunofluorescence Staining of Mouse Brain Tissues

Mouse brains were first fixed with 4% PFA at 4°C for 24 hours, and the brains were later cryoprotected in 15% and 20% sucrose in PBS for 24 hours at 4°C. Coronal sections (40 *μ*m) were cut in a crystal. Sections were blocked with goat serum containing Triton X-100 for 2 h at room temperature and then incubated with rabbit BDNF antibodies (1:500 dilution; Abcam; ab108319) overnight at 4°C. Sections were washed and later incubated with a 1:500 dilution of Alexa 488-conjugated goat antirabbit antibodies for 1 h at room temperature.

### 2.7. Western Blot

The separated striatum and cells were homogenized in RIPA lysis buffer with Protease Inhibitor Cocktail at 4°C and quantified using the BCA protein assay kit (Beyotime Biotechnology). Sixty micrograms of protein was separated via sodium dodecyl sulfate polyacrylamide gel electrophoresis and was transferred to PVDF membranes (Millipore). Immunolabeling was performed using rabbit BDNF antibodies (1:1000 dilution; Abcam; ab108319), mouse IP_3_R_2_ antibodies (1:1000 dilution; Santacruz; sc398434), mouse monoclonal actin antibodies (1:400 dilution; BOSTER; BM0627), and rabbit monoclonal tubulin antibodies (1:1000 dilution; BOSTER; BM3885). The results were visualized by enhanced chemiluminescence (GE Healthcare Bio-Science, Uppsala, Sweden). Images were captured and documented with a charge-coupled device system (Image Station 2000MM; Kodak, Rochester, NY, USA). Quantitative analysis of the images was performed using Molecular Imaging Software (version 4.0, as part of the Kodak 2000MM System).

### 2.8. Statistical Analysis

The data were analyzed using the SPSS statistical software package (version 17.0; IBM Corp., Armonk, NY, USA). Mean values were compared using unpaired *t*-test; Welch's correction is used when variance is unequal. *P* values <0.05 were considered statistically significant. The voxel-wise statistical comparisons of the GM images and FC maps were done using SPM12. A two-sample *t*-test was adopted for the between-group comparisons with a significance threshold of *P* < 0.01 (with AlphaSim correction of *P* =0.05).

## 3. Results

### 3.1. Itpr2^−/−^ Mice Exhibit Depressive-Like Behaviors

**Figure 1:**
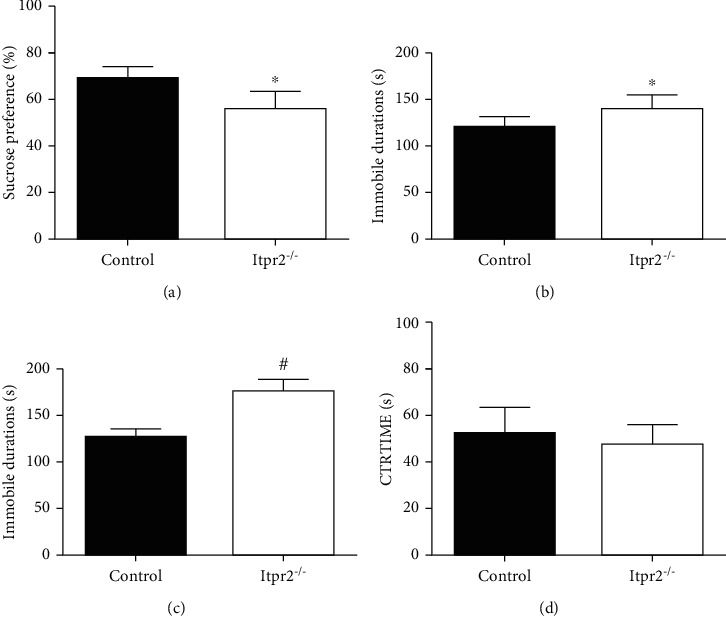
Behavior test of *Itpr2^−/−^* mice and controls. (a) Sucrose preference. (b) Tail suspension test immobile time. (c) Forced swim test immobile time. (d) Open-field test center time. Data are expressed as the mean ± SD. Asterisk indicates *P* < 0.05 and number sign indicates *P* < 0.01 when compared with the controls.

We firstly tested whether *Itpr2*^−/−^ mice exhibited depressive-like behaviors by performing behavioral tests on both the *Itpr2*^−/−^ mice and controls. Results revealed that the *Itpr2*^−/−^ mice exhibited less sucrose consumption (*P* = 0.017, *P* < 0.05, *t* = 2.920, df = 9, *F* = 1.817) (Figure 1(a)) and increased duration of immobility both in tail suspension test (*P* = 0.0385, *P* < 0.05, *t* = 2.381, df = 10, *F* = 1.943) (Figure 1(b)) and forced swim test (*P* < 0.0001, *t* = 7.652, df = 10, *F* = 1.900) (Figure 1(c)) compared with controls. In the open-field test, we firstly analyzed the movement speed of the two groups of mice to rule out the effect of locomotor ability on behavior performance, and there was no significant difference between the two groups (Supplementary materials Figure 1). Although there was a decreased central retention time in *Itpr2*^−/−^ mice, the difference was not statistically significant (*P* = 0.4602, *t* = 0.7758, df = 8, *F* = 1.643) (Figure 1(d)).

### 3.2. Reduced Volume of Gray Matter in Itpr2^−/−^ Mice

**Figure 2:**
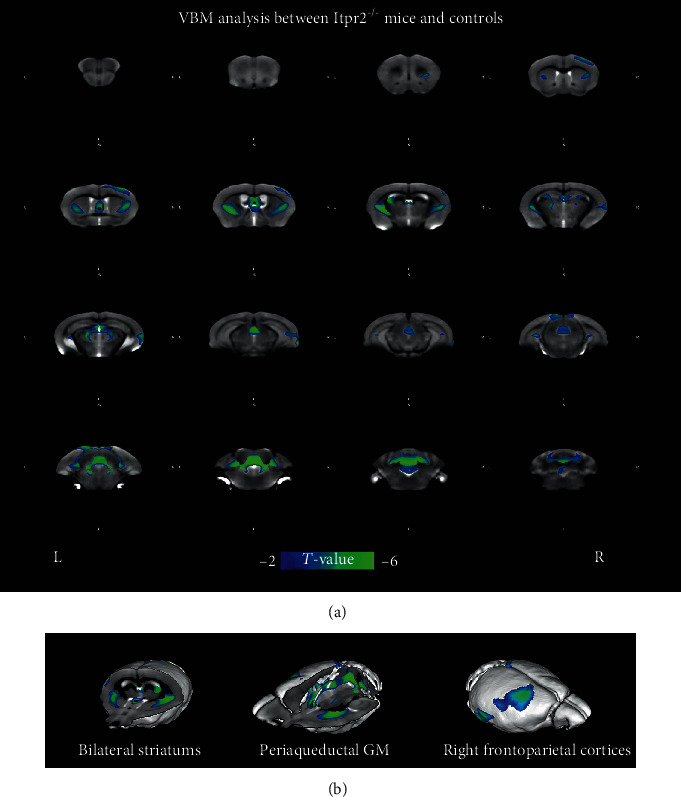
Abnormal volume of the gray matter in *Itpr2^−/−^* mice as compared with controls. (a) The regions of decreased volume of gray matter shown on the coronal plane. (b) The regions of decreased volume of gray matter shown on the three-dimensional images.

VBM analysis showed that the *Itpr2*^−/−^ mice group was found to have significantly reduced gray matter volumes in bilateral striatums, the periaqueductal gray matter, and the right frontoparietal cortices compared with the control group (*P* < 0.01, AlphaSim corrected) (Figure 2), whereas no region was found with significantly increased gray matter volume in *Itpr2*^−/−^ mice.

### 3.3. Abnormal Functional Connectivity Related to the Striatum in Itpr2^−/−^ Mice

**Figure 3:**
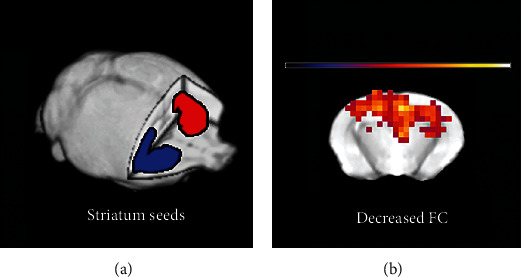
Abnormal resting-state functional connections between other regions of the whole brain with the bilateral striatum in both groups. Whole-brain RSFC map is created based on the blood oxygen level-dependent time series from spherical seed regions of the striatum. (a) Positions of striatum seeds. (b) Decreased FC between the striatums and other brain regions in Itpr2^−/−^ mice.

Based on the results in Section 3.2, the striatum was selected as seed ROI for further FC analysis (Figure 3(a)), since this region was considered highly relevant to the emotion regulation and depression in previous studies. The RSFC maps of the bilateral striatums were highly relevant to each other. Therefore, to limit the number of statistical tests and for easy interpretation of the results, the RSFC maps of the bilateral striatums were averaged for each mouse and subsequently compared between groups. Compared with controls, significantly decreased striatal FC was found in the bilateral hippocampus and right parietal cortex (mainly for the primary and secondary somatosensory cortex) in the *Itpr2*^−/−^ mice (*P* < 0.01, AlphaSim corrected); however, no region was identified with increased FC (Figure 3(b)).

### 3.4. Decreased Expression of BDNF in the Striatums of Itpr2^−/−^ Mice and Itpr2^−/−^ C8-D1A

**Figure 4:**
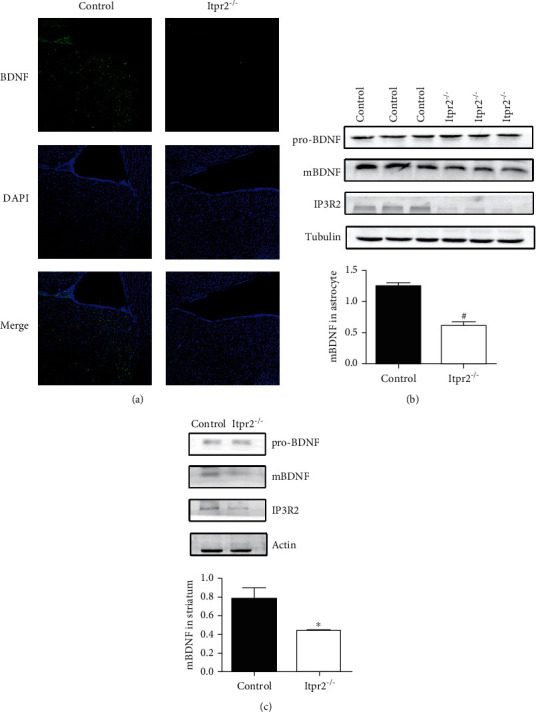
Detection of BDNF level in *Itpr2^−/−^* samples and controls. (a) BDNF immunofluorescence staining and expression in the striatum (×100). (b) Analysis of BDNF expression in the striatum by Western blotting. (c) Analysis of BDNF expression in C8-D1A by Western blotting. Data are expressed as the mean ± SD. Number sign indicates *P* < 0.01 when compared with the controls.

Immunofluorescence staining showed a declined level of BDNF in the striatums of *Itpr2^−/−^* mice (Figure 4(a)). And the striatums of both *Itpr2^−/−^* mice and controls (Figure 4(b)), as well as cells including *Itpr2^−/−^ C8-D1A* and controls (Figure 4(c)), were later subjected to Western blotting using anti-BDNF antibodies. In all samples tested, we detected 14- and 28 kDa BDNF-immunoreactive bands representing pro- and mature BDNF, respectively. The intensity of the pro-BDNF band was not significantly different between *Itpr2^−/−^* samples and controls both in mice and astrocytes, while the expression of mature BDNF decreased clearly in the *Itpr2^−/−^* sample as compared with controls both in mice (*P* = 0.0368, *P* < 0.05, *t* = 5.067, df = 2, *F* = 653.0) and astrocytes (*P* = 0.002, *P* < 0.01, *t* = 13.04, df = 4, *F* = 1.068).

## 4. Discussion

This paper confirmed the previous finding that *Itpr2^−/−^* mice exhibit depressive-like symptoms as shown in most behavioral tests except in OFT. However, OFT is mainly used to test whether the mice perform obvious anxiety and exploratory intentions, which suggests *Itpr2^−/−^* mice did not perform these symptoms. On this basis, we further revealed *in vivo* brain abnormalities including a reduced striatum volume and relevant functional connectivity in *Itpr2^−/−^* mice. Additionally, to further study the *in vivo* MR findings, we performed biochemistry analyses and found decreased BDNF expression in both the sample tissues of the striatum in the *Itpr2^−/−^* mice and *Itpr2^−/−^* C8-D1A. Thus, these findings may jointly support the relationship between the genetic deficiency of Itpr2 and the currently observed *in vivo* brain abnormalities.

As one of the crucial roles of reward and emotion regulation networks, the striatum receives cortical and dopaminergic projections; it is located at the center of functional circuits that influence motor and cognitive aspects of behavior [25–27] and has been frequently discussed in studies of emotion regulation strategies and was found to contribute directly to decision-making [28, 29]; the relationship between depression and the striatum is extensively studied. Previous research reported volumetric abnormalities of the striatum or part of the striatums such as the lentiform nucleus in major depression disorder (MDD) patients. Matsuo et al. have indicated that treatment-naive MDD patients have significantly smaller right striatum and right caudate (part of the striatum) volumes compared to the healthy subjects [30], suggesting that depression closely depends on the structure and functioning of the striatum [31–34].

Reduction of BDNF in the brain has been proposed as a candidate for possible involvement in depression [35]. Previous studies indicated that increased levels of BDNF in the striatum is related to improved depressive-like behavior [36]. Moreover, this study found the decreased expression of mature BDNF in the striatum, which possibly contributes to the reduced volume of the striatum and depressive-like behaviors in *Itpr2^−/−^* mice; this is since, decreased BDNF can lead to tissue loss.

As mentioned above, IP_3_R_2_ is mainly expressed in astrocytes in the brain and BDNF is mainly generated and released by astrocytes. For this reason, we further detected the expression level of BDNF in astrocytes lacking Itpr2 (*Itpr2^−/−^* C8-D1A) and found it decreased. So, being consistent with our findings in tissue, this result supported that the decline of BDNF in the striatums in *Itpr2^−/−^* mice is induced by the reduced BDNF expression of astrocyte lacking Itpr2.

The strength of memory generated by the hippocampus is related to the level of FC between the hippocampus and the striatum [37]. Previous research has revealed that the FC between the hippocampus and the striatum increased significantly after a period time of memory training, promoting the changed FC is responsible for the formation of long-term memories [38]. Our results suggest the decreased FC the hippocampus and the striatum can lead to the weakened ability of memory which is a common symptom in depression. Besides, lower FC between the striatum and right parietal cortex (mainly for the primary and secondary somatosensory cortex) may participate in the painful physical symptoms which are usually present in depression.

MRI as a noninvasive tool can provide clues about *in vivo* functional connectivity and volume abnormalities, which were then combined with biochemical analyses to validate and explain the possible pathogenesis in *Itpr2^−/−^* mice from the perspective of dysfunctional astrocytes. Such kind of combined studies is of some value, especially for the psychiatric disorders like depression, since it may shed light on the neuropathological mechanism from the perspective of *in vivo* neural abnormalities, and could be a strength of this work. However, in this paper, we did not further study the specific mechanism by which the absence of Itpr2 leads to the decreased expression of BDNF in astrocyte, and we also lack research on the learning and memory abilities of mice related to decreased FC of striatums-hippocampus and painful physical symptoms related to decreased FC of striatums-right parietal cortex. So, in the future, we should go on to study the underlying mechanism in astrocytes lacking Itpr2 and supplement other experiments to complete the study.

## 5. Conclusion

By combining biochemistry and MR analyses, this study revealed that the Itpr2-related neuropathological effect is possibly mediated by the relevant brain especially striatum structural and functional abnormality in *Itpr2*^−/−^ mice *in vivo*, which is associated with decreased BDNF in the striatum due to a decline production of BDNF from astrocyte lacking Itpr2; thus, it may help us better understand underlying mechanisms of Itpr2 deficiency as well as its relation to depressive behavior.

## Data Availability

The datasets used to support the findings of this study are available from the corresponding author on reasonable request.

## Supplementary Materials

Supplementary Materials Figure 1. Movement speed of the Itpr2^−/−^ and control group in OF.
