# Supplementary figures and images for: In Vivo Structural and Functional Abnormalities of the Striatums Is Related to Decreased Astrocytic BDNF in Itpr2−/− Mice Exhibiting Depressive-Like Behavior
Source: Neural Plast. 2020 Sep 1;2020:8830670. doi: 10.1155/2020/8830670 (PMC7481938; doi:10.1155/2020/8830670)

## Supplementary materials

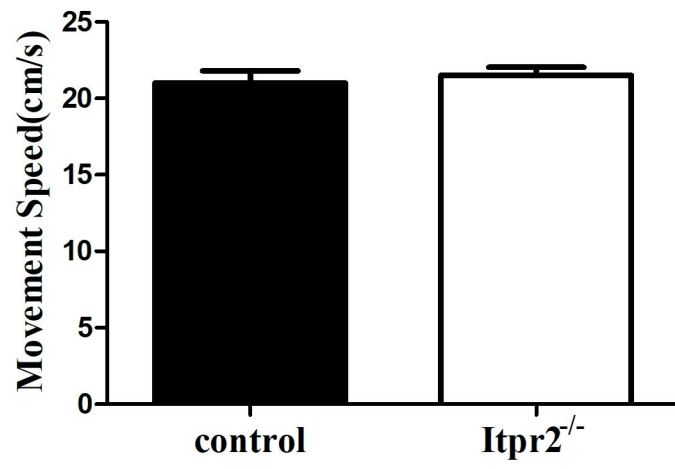

FIGURE 1. Movement speed of *Itpr2*<sup>-/-</sup> and control group in OFT.

Supplement: Supplementary Materials — Figure 1. Movement speed of the Itpr2−/− and control group in OF. [file 8830670.f1.pdf]
